# Spectral- and time-domain optical coherence tomography measurements of macular thickness in young myopic eyes

**DOI:** 10.1186/1746-1596-9-38

**Published:** 2014-02-20

**Authors:** Lin Liu, Jun Zou, Lili Jia, Jian-guo Yang, Shao-rong Chen

**Affiliations:** 1 Department of Ophthalmology, Shanghai Jiaotong University Affiliated Sixth People’s Hospital, Shanghai 200233, China

**Keywords:** Myopia, Macular retinal thickness, Optical coherence tomography, Time-domain, Spectral-domain

## Abstract

**Purpose:**

To evaluate the variation in macular retinal thickness and volume in young Chinese myopic patients using time-domain optical coherence tomography (Stratus TD-OCT) and spectral-domain optical coherence tomography (Cirrus HD-OCT).

**Methods:**

Ninety-two eyes of 92 myopic subjects were recruited in this study. Based upon spherical equivalence (SE), subjects were divided into two groups: the low to moderate myopia group (-0.5 D ≤ SE < -6.0 D), and the high myopia group (SE ≥ -6.0 D). Stratus TD-OCT and Cirrus HD-OCT were used to compare macular retinal thickness and volume between the two groups. Bland–Altman analysis and Pearson correlation were used to measure agreement between the two OCT systems.

**Results:**

Average macular retinal thickness and total macular volume measured by Cirrus HD-OCT and Stratus TD-OCT of the low to moderate myopia group were 283.52 ± 12.14 μm and 245.38 ± 8.55 μm, respectively, and 10.08 ± 0.37 mm^3^ and 6.85 ± 0.26 mm^3^, respectively, and the high myopia groups were 269.58 ± 10.72 μm and 235.65 ± 7.54 μm, respectively, and 9.71 ± 0.36 mm^3^ and 6.52 ± 0.25 mm^3^, respectively. The measurements of the two OCTs showed that macular retinal thickness of the parafovea was significantly lower in the high myopia group compared with the low to moderate myopia group, except at the fovea (all P-values less than 0.001, except at the fovea). Using the Bland–Altman method and Pearson correlation, measurements of macular thickness in nine macular retinal subfields and total macular volumes showed good agreement between the two OCTs in myopic eyes (all P-values less than 0.001), with better agreement in the low to moderate myopia group than in the high myopia group.

**Conclusions:**

The average macular retinal thickness of the fovea did not vary with myopia, while the total volume and retinal thickness of the parafovea were thinner with increasing myopia. There was good agreement between the two OCTs in myopic eyes in all macular subfields, and the Cirrus HD-OCT system provided thicker macular retinal thickness measurements than the Stratus TD-OCT system.

**Virtual slides:**

The virtual slides for this article can be found here: http://www.diagnosticpathology.diagnomx.eu/vs/1683223414107652.

## Background

In eastern Asia, myopia has reached epidemic proportions, and the prevalence of myopia has continued to increase [1-3]. High myopia is one of the commonest types of human ocular optical disorders, which can result in complications such as cataract, posterior scleral staphyloma, retinal detachment, choroidal neovascularization, and macular holes [4-8]. The retina can degenerate and become thinner in myopic eyes, especially at the posterior pole [9], so characterizing the associations between macular thickness of myopia and macular diseases has important clinical implications.

Optical coherence tomography (OCT) is a noninvasive, cross-sectional imaging technique that can measure macular thickness, and is highly reproducible [10-12]. Apart from its diagnostic value in uveitis, OCT has enabled objective assessment of treatment response, provided predictive value for visual recovery and prognosis of uveitic entities, and detected toxic effects of the drug [13,14]. The purpose of this study was to evaluate the variations in macular retinal thickness and volume in young Chinese myopic patients by using time-domain optical coherence tomography (Stratus TD-OCT) and spectral-domain optical coherence tomography (Cirrus HD-OCT), to identify the morphological features of the macula in macular diseases. These techniques could evaluate risk factors associated with myopia, which may assist in early diagnosis and monitoring of macular changes.

## Material and methods

### Subjects

Ninety-two eyes of 92 consecutive refractive surgery candidates with spherical equivalents ≥ -0.5 diopters (D) were recruited for the study. Liu et al. have reported [15] corneal astigmatism effects on the OCT measurements among high myopes, therefore corneal astigmatism of each subject was less than -1.0 D, especially in the high myopia group. The study has been approved by the Institutional Ethnic Committee (Institutional Review Board) of Shanghai Jiaotong University Affiliated Sixth People’s Hospital, and informed consent was obtained for each patient.

Each subject underwent a full ophthalmic examination, which included measurements of visual acuity, refraction, and intraocular pressure (IOP) by a noncontact tonometer. Axial length measurements were obtained in each eye with the IOL Master (Carl Zeiss Meditec, Inc, Dublin, CA, USA). Optic nerve head evaluation was performed with a 90-D lens. Macular retinal thickness and volume were measured by a Stratus TD-OCT and Cirrus HD-OCT (Carl Zeiss Meditec, Dublin, CA, USA).

Inclusion criteria were as follows: best corrected visual acuity (BCVA) of 20/20 or better, an IOP lower than 21 mm Hg in either eye, a healthy optic nerve head (ONH) without glaucomatous damage (i.e., no disc hemorrhage, notching, or thinning of the neural rim).

Those with a history of severe ocular trauma, intraocular or refractive surgery, or any ocular or neurological disease that could have affected the ONH or retinal nerve fiber layer (RNFL) were excluded from the study. Subjects with evidence of macular disease or peripapillary atrophy extending more than 1.73 mm from the center of the optic disc or with glaucoma or an IOP higher than 21 mmHg in either eye were also excluded. In addition, participants with a history of systemic diseases including hypertension and diabetes were excluded.

### OCT imaging

After pupillary dilation to a minimum diameter of 5 mm, eyes of the subjects that satisfied the study criteria were scanned using the Cirrus HD-OCT system with software version 5.0 and Stratus TD-OCT system with software version 4.0. The mean retinal thickness and volume maps were determined for nine sectors in three concentric circles of diameters 1, 3, and 6 mm. The inner and outer rings were divided into four quadrants as follows: outer of temporal (To), outer of superior (So), outer of inferior (Io), outer of nasal (No), inner of temporal (Ti), inner of superior (Si), inner of inferior (Ii), and inner of nasal (Ni). The foveal thickness (F) was defined as the average thickness in the central 1 mm diameter. All the scans had signal strength of at least six and all measurements were taken by a single, trained examiner.

### Statistical analysis

Statistical analyses were performed with commercially available software (SPSS ver. 12.0; SPSS Inc, Chicago, IL, USA). The total average and mean macular retinal thickness and macular volume were compared between the two groups with an independent *t*-test (except for the gender, which was compared by the chi-square test). Agreements between the two OCT systems were measured by Bland–Altman method and the Pearson correlation. A P-value less than 0.05 was considered statistically significant.

## Results

### Patient characteristics

Of ninety-two eyes, the mean age of the subjects was 22.89 ± 3.27-years-old, the average spherical equivalent (SE) was -5.64 ± 2.66 D (diopters) with a range of -0.38 D to -11.00 D. Based upon spherical equivalence (SE), subjects were divided into two groups: the low to moderate myopia group (-0.5 D ≤ SE < -6.0 D), and the high myopia group (SE ≥ -6.0 D). Forty-four eyes were classified as low to moderate myopia group with a mean SE of -3.34 ± 1.28 D, and 48 eyes were classified as high myopia group with an average SE of -7.75 ± 1.65 D. Characteristics of the two groups are listed in Table 1. No significant differences were found for age, sex, axial length, and spherical equivalence between the two groups.

**Table 1:** **Characteristics of the LMM and HM groups**$\left(\bar{x}\pm s\right)$

	**Sex (male/female)**	**Eye (R/L)**	**Age (y)**	**Spherical equivalent(D)**	**Axial length (mm)**
LMM group	24/20	25/19	22.20 ( 2.98)	3.34 ( 1.28)	24.86 (1.09)
HM group	20/28	23/25	23.02 (3.32 )	7.75 ( 1.65)	26.78 (0.97)
*χ*2 / F value	*χ*2 = 1.526	*χ*2 = 0.729	F = 2.22	F = 0.73	F = 1.148
*P* value	0.217^a^	0.393^a^	0.219^b^	<0.001^b^	<0.001^b^

^a^*χ2* test, ^b^Independent *t*-test; LMM group, low to moderate myopia group; HM group, high myopia group; y, years; D, diopters.

### Topographic profile of macular retinal thickness among young myopic patients

From Table 2, the average macular retinal thickness and total macular volume measured by Cirrus HD-OCT and Stratus TD-OCT of the low to moderate myopia group were 283.52 ± 12.14 μm and 245.38 ± 8.55 μm, 10.08 ± 0.37 mm^3^ and 6.85 ± 0.26 mm^3^, respectively, and of the high myopia group were 269.58 ± 10.72 μm and 235.65 ± 7.54 μm, 9.71 ± 0.36 mm^3^ and 6.52 ± 0.25 mm^3^, respectively. Measurements using the two OCTs showed that the macular retinal thickness of the parafovea was significantly lower in the high myopia group compared with the low to moderate myopia group, except at the fovea (all P-values less than 0.001, except at the fovea).

**Table 2:** Comparison of macular thickness and volume as determined by optical coherence tomography (OCT)

	**Low to moderate myopia group**		**High myopia group**		***P***_***1***_	***P***_***2***_
	**Cirrus HD OCT**	**Stratus TD OCT**	**Cirrus HD OCT**	**Stratus TD OCT**		
Avg	283.52 (12.14)	245.38 (8.55)	269.58 (10.72)	235.65 (7.54)	0.000	0.000
F	251.16 (15.20)	195.89 (14.43)	250.08 (16.65)	192.35 (14.99)	0.748	0.254
Ti	309.82 (13.49)	257.68 (12.66)	301.31 (9.90)	250.67 (8.76)	0.001	0.003
Si	324.43 (12.90)	273.00 (11.84)	315.42 (10.53)	264.94 (7.99)	0.000	0.000
Ni	323.57 (13.18)	272.30 (12.62)	315.46 (10.01)	262.44 (8.88)	0.001	0.000
Ii	316.86 (11.66)	267.52 (11.55)	307.85 (11.38)	259.96 (9.64)	0.000	0.001
To	264.80 (12.60)	218.52 (9.11)	253.00 (14.83)	204.85 (11.80)	0.000	0.000
So	283.77 (12.00)	241.43 (12.76)	272.19 (11.85)	228.81 (11.59)	0.000	0.000
No	306.84 (10.90)	260.57 (12.66)	290.77 (13.82)	246.67 (12.66)	0.000	0.000
Io	267.25 (10.90)	221.55 (10.52)	254.10 (11.42)	210.17 (11.09)	0.000	0.000
Vol	10.08 (0.37)	6.85 (0.26)	9.71 (0.36)	6.52 (0.25)	0.000	0.000

*P*_*1,*_ Comparison of macular thickness and volume measured by Cirrus HD-OCT.

*P*_*2,*_ Comparison of macular thickness and volume measured by Stratus TD-OCT.

### Correlations between macular retinal thickness, macular volume, and axial length

The change of axial length is a characteristic of myopic eyes, so the morphological features of the macula may change with axial length. As shown in Table 3, the average macular retinal thickness of the parafovea and macular volume decreased with axial length, (all P-values less than 0.001, except at the fovea), especially in outer parafovea retinal subfields. These results demonstrated that with increase of axis length, macular thickness became thinner.

**Table 3:** Correlation analyses between axial length and retinal thickness/volume

	**Cirrus HD OCT**		**Stratus TD OCT**	
	**r**_**1**_	***P***_***1***_	**r**_**2**_	***P***_***2***_
Avg	-0.361	0.000	-0.432	0.000
F	0.127	0.227	0.087	0.412
Ti	-0.260	0.012	-0.226	0.032
Si	-0.212	0.043	-0.317	0.002
Ni	-0.163	0.039	-0.225	0.033
Ii	-0.278	0.007	-0.287	0.006
To	-0.365	0.000	-0.553	0.000
So	-0.387	0.000	-0.416	0.000
No	-0.469	0.000	-0.457	0.000
Io	-0.534	0.000	-0.562	0.000
Vol	-0.326	0.001	-0.532	0.000

Pearson analysis, *n* = 92.

### Comparisons of OCT measurements of macular thickness and macular volume

**Figure 1:**
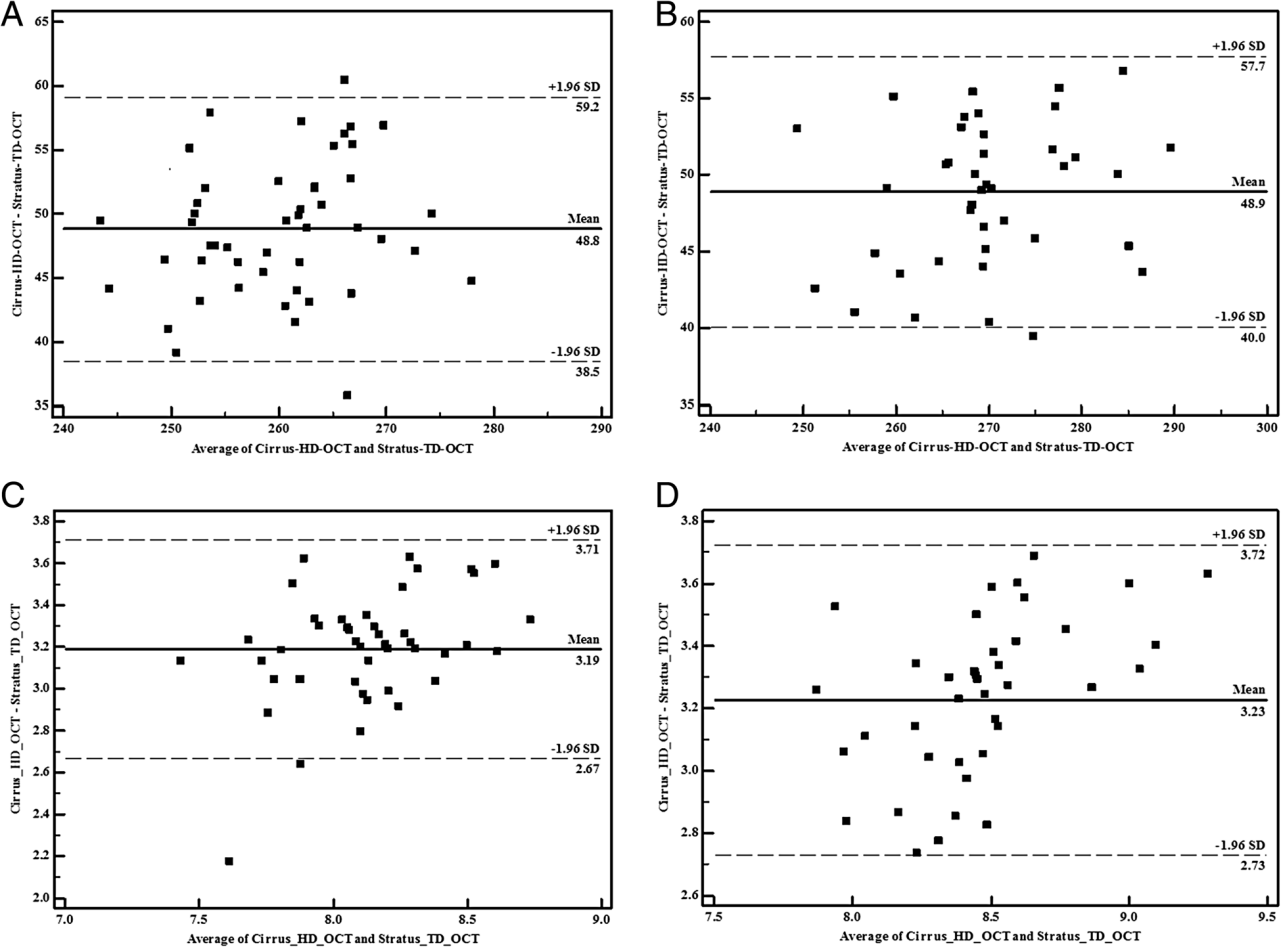
**Bland–Altman plots of average macular retinal thickness/macular volume values between Stratus TD-OCT and Cirrus HD-OCT. (A)** Average macular retinal thickness in the low to moderate myopia group; **(B)** average macular retinal thickness in the high myopia group; **(C)** total macular volume in the low to moderate myopia group; **(D)** total average macular volume in the high myopia group. The difference (Cirrus HD-OCT-Stratus/TD-OCT) is plotted against the average of both measurements (Cirrus HD-OCT + Stratus TD-OCT/2) for all participants in each group. SD, standard deviation.

Table 2 and Table 4 show that the Cirrus HD-OCT system provided thicker macular retinal thickness measurements than the Stratus TD-OCT system. The measurements of macular thickness in nine macular retinal subfields and total macular volume showed good agreement between the two OCTs in myopic eyes in all macular subfields, using the Bland–Altman method and Pearson correlation (all P-values less than 0.001), with a better agreement in the low to moderate myopia group than in the high myopia group (Figure 1).

**Table 4:** Bland-Altman analysis of macular thickness and volume values between Stratus TD-OCT and Cirrus HD-OCT

**Low to moderate myopia group**					**High myopia group**			
**Mean differences**		**The 95% agreement limit**	**Range**	**1.96 × SD**	**Mean differences**	**The 95% agreement limit**	**Range**	**1.96 × SD**
Avg	48.89 ± 5.28	40.04-57.75	17.71	10.35	48.82 ± 4.52	38.47-59.16	20.69	8.86
F	55.27 ± 6.35	42.83-67.71	24.88	12.45	57.73 ± 7.64	42.76-72.70	29.94	14.97
Ti	52.13 ± 7.34	37.75-66.52	28.77	14.39	50.65 ± 6.59	37.74-63.56	25.82	12.92
Si	51.43 ± 6.33	39.02-63.85	24.83	12.41	50.48 ± 7.10	36.57-64.39	27.82	13.92
Ni	51.27 ± 9.22	33.19-69.35	36.16	18.07	53.02 ± 8.11	37.11-68.93	31.82	15.90
Ii	49.34 ± 6.80	36.02-62.66	26.64	13.33	47.90 ± 7.15	33.89-61.90	28.01	14.01
To	46.27 ± 8.39	29.82-62.71	32.89	16.44	48.15 ± 11.38	25.83-70.46	44.63	22.30
So	42.34 ± 8.72	25.26-59.42	34.16	17.09	43.38 ± 8.29	27.13-59.62	32.49	16.25
No	46.28 ± 7.97	30.65-61.90	31.25	15.62	44.10 ± 6.64	31.08-57.13	26.05	13.01
Io	45.70 ± 5.95	34.05-57.36	23.31	11.66	43.94 ± 6.34	31.51-56.37	24.86	12.43
Vol	3.23 ± 0.25	2.73-3.72	0.99	0.49	3.19 ± 0.27	2.67-3.71	1.04	0.52

## Discussion

Myopia is one of the most common causes of visual impairment and blindness, so early detection and management of degenerative eye diseases is of utmost importance. The results of the current study demonstrated that average macular retinal thickness of the fovea did not vary with myopia, while total volume and the retinal thickness of the parafovea were thinner with increased myopia. There was good agreement between the two OCTs in myopic eyes in all macular subfields, and the Cirrus HD-OCT system provided thicker macular retinal thickness measurements than the Stratus TD-OCT system.

Previous studies reported that Cirrus HD-OCT measurements resulted in a significantly thicker retinal thickness than the Stratus TD-OCT [16-18]. We also found that Cirrus HD-OCT measurements resulted in thicker retinal thickness measurements than the Stratus TD-OCT in myopic eyes. Different technologies and segmentation algorithms of the two OCT systems may explain the thicker retinal thickness measurements observed in Cirrus HD-OCT. The most important difference is that the Cirrus segmentation identified the thickness of the retina from the retinal pigment epithelium (RPE) to the internal limiting membrane (ILM), while the Stratus segmentation identified retinal thickness from the inner segment/outer segment junction (IS/OS) to the ILM, thus Cirrus HD-OCT measurements resulted in an inherently thicker distance between the IS/OS and the RPE [19].

Our study showed that the two OCTs showed macular retinal thickness of the parafovea, especially in outer retinal subfields, that was significantly lower in the high myopia group compared with the low to moderate myopia group, except at the fovea (all P-values less than 0.001, except at the fovea). Other studies demonstrated that the average macular retinal thickness of the parafovea and total macular volume decreased with axial length (all P-values less than 0.001, except at the fovea). So with an increase of axis length, macular thickness becomes thinner, especially in the outer retinal subfields. Marcus et al. and Lim et al. also reported that the parafovea was thinner in myopic patients’ myopia [20]. This observation may be the result of the anatomical features of the retinal macular foveolar region. The foveolar is the thinnest area of the retina, which consists of the five innermost cell layers of the retina, including the outer plexiform layer, the outer nuclear layer, the external limiting membrane, the photoreceptor layer, and the retinal pigment epithelium. Previous histopathologic studies reported that the thickness of neurosensory retina (RNL), especially the inner nuclear layer, were thinner in several animal models of myopia [21]. The foveolar lacks the innermost retinal layers, and there is no inner nuclear, so the retinal thickness variation is not obvious between the high myopia group and moderate myopia groups. Therefore, the macular retinal thickness in the parafovea was thinner in the high myopia group than in the moderate myopia group, but no difference was found in the foveolar. Moreover, other pathological processes, such as choroid retinal degeneration, and choroid and RPE atrophy, could likewise play a role in changes of the retina.

The results of the current study characterized macular retinal thickness in young myopic patients. These characteristics can be used as an auxiliary diagnosis for making a distinction with other macular retinal related diseases. If the macular thickness in the foveolar is abnormally thin, pathological disease should be considered. However, if the changes occurred in the parafovea, other factors can first be eliminated, such as refractive factors, which could influence the measurements of macular thickness by the OCTs, especially among high myopia patients. In conclusion, when the macular thickness is abnormal, clinicians should consider the effect of the refractive factor combined with clinical manifestations to comprehensively evaluate its clinical significance.

## Competing interests

The authors declare that they have no competing interests.

## Authors’ contributions

LL participated in the study design, reviewed the literature, collected the clinical data, and drafted the manuscript. JZ provided the conception and design of the study and reviewing the manuscript. L-lJ collected the clinical data and selected the material. J-gY took part in the study design and performed the statistical analysis. S-rC participated in collected the clinical data. All authors have read and approved the manuscript.
